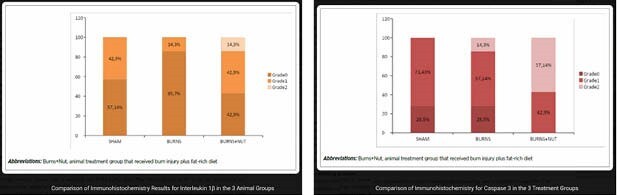# 109 Effects of Fat-Rich Nutrients in the Pancreas of an Experimental Burn Model

**DOI:** 10.1093/jbcr/irad045.082

**Published:** 2023-05-15

**Authors:** Santiago Santelis, Ayse Ebru Abali, Neslihan Basci Tutuncu, B Handan Ozdemir, Gonca Ozgun, Nilufer Bayraktar, Meric Yavuz Colak, Mehmet Haberal

**Affiliations:** Burn Center and Burn and Fire Disasters Institute, Baskent University, Sanford, Florida; Baskent University, Ankara, Ankara; Baskent University, Ankara, Ankara; Baskent University, Ankara, Ankara; Baskent University, Ankara, Ankara; Baskent University, Ankara, Ankara; Baskent University, Ankara, Ankara; Baskent University, Ankara, Ankara; Burn Center and Burn and Fire Disasters Institute, Baskent University, Sanford, Florida; Baskent University, Ankara, Ankara; Baskent University, Ankara, Ankara; Baskent University, Ankara, Ankara; Baskent University, Ankara, Ankara; Baskent University, Ankara, Ankara; Baskent University, Ankara, Ankara; Baskent University, Ankara, Ankara

## Abstract

**Introduction:**

Oversupply of nutrients overstimulates beta cells in standard conditions, and severe burn injuries increase the metabolic needs. In this study, we investigated the effects of fat-rich nutrients on the endocrine pancreas during the acute phase of severe burns.

**Methods:**

Twenty-one Wistar albino rats were randomly divided into 3 groups. Two groups were fed with standard commercial rat chow (1 with sham procedure and 1 with burn procedure), and the third group (also with burn procedure) was fed a fat-rich diet (60% kcal fat). The burn procedure involved a 25% total body surface area fullthickness burn. Blood samples were taken at 36 hours and 7 days after burn injury or sham procedure. On postburn day 7, skin biopsies were taken and a pancreatectomy was performed. Pancreatic tissues were examined under light microscopy; islets size and cellularity were calculated and investigated immunohistochemically.

**Results:**

Plasma glucose, C-Peptide, and insulin levels were similar in all the study groups 36 hours and 7 days after burn induction or sham procedure. There was a significant increase in the number of cells per one islet in the burn group given a fat-rich diet compared with the other groups (P = .05). Caspase-3 was strongly expressed in both groups with burn injuries.

**Conclusions:**

Overconsumption of certain fats can lead to a compensatory response by beta cells that eventually can progress to beta-cell dysfunction during the acute phase of burns. Severe burns induce pancreatic islet cell hyperplasia. Providing excessive fat nutrients during the acute postburn period attenuates this response. Despite the morphophysiological changes observed in the pancreas, all animals in our study achieved similar glycemic homeostasis.

**Applicability of Research to Practice:**

The delivery of nutritional support is a vital element of burn care, and the main goal is simply to avoid nutritional complications. Lipotoxicity contributes to multisystem organ failure and worse clinical outcomes in obese patients and mice with pancreatitis. Neutralization of IL-1β produces beneficial effects on postburn glycemic control and improves survival. apoptosis in the pancreas of patients with severe burns may be linked to a series of events that can induce Caspase-1 activation and IL-1β release, and these pathways may be influenced by the nutritional status, especially in the early postburn phase. Effective nutritional assessment and management can optimize wound healing and decrease complications and mortality.